# Perspectives of Genome Editing Mediated Haploid Inducer Systems in Legumes

**DOI:** 10.3390/ijms26031154

**Published:** 2025-01-29

**Authors:** Yiqian Liu, Musazade Elshan, Geng Li, Xiao Han, Xiao Chen, Xianzhong Feng

**Affiliations:** 1 Agronomy College, Jilin Agricultural University, Changchun 130118, China; liuyiqian@mails.jlau.edu.cn (Y.L.); hanxiaoyy@jlau.edu.cn (X.H.); 2 Key Laboratory of Soybean Molecular Design Breeding, National Key Laboratory of Black Soils Conservation and Utilization, Northeast Institute of Geography and Agroecology, Chinese Academy of Sciences, Changchun 130102, China; elshan.musazade1@gmail.com (M.E.); ligenghap@163.com (G.L.); 3 College of Life Sciences, Jilin Agricultural University, Changchun 130118, China

**Keywords:** haploid inducer lines, haploid production, genome editing, legume breeding

## Abstract

Genome editing-mediated haploid inducer systems (HISs) present a promising strategy for enhancing breeding efficiency in legume crops, which are vital for sustainable agriculture due to their nutritional benefits and ability to fix nitrogen. Traditional legume breeding is often slow and complicated by the complexity of legumes’ genomes and the challenges associated with tissue culture. Recent advancements have broadened the applicability of HISs in legume crops, facilitating a reduction in the duration of the breeding cycle. By integrating genome editing technology with haploid breeding systems, researchers can achieve precise genetic modifications and rapidly produce homozygous lines, thereby significantly accelerating the development of desired traits. This review explores the current status and future prospects of genome editing-mediated HISs in legumes, emphasizing the mechanisms of haploid induction; recent breakthroughs; and existing technical challenges. Furthermore, we highlight the necessity for additional research to optimize these systems across various legume species, which has the potential to greatly enhance breeding efficiency and contribute to the sustainability of legume production.

## 1. Introduction

Legume crops, including soybeans (*Glycine max*), peas (*Pisum sativum*), lentils (*Lens culinaris*), and chickpeas (*Cicer arietinum*), are vital for global food security, nutrition, and sustainable agriculture. Soybeans, in particular, serve as a crucial food source worldwide, providing valuable plant-based protein and contributing to soil health through nitrogen fixation. However, legume breeding faces challenges due to legumes’ large, complex genomes, coupled with significant genetic diversity. Traditional breeding methods often cannot keep pace with the increasing demand for higher yields and improved crop quality. These methods encounter obstacles such as lengthy breeding cycles, sexual incompatibility, and fertilization barriers, which limit their efficiency [1]. Although traditional breeding can introduce desirable traits, it may inadvertently reduce genetic diversity, rendering crops more susceptible to environmental stresses and failing to address pressing global food security issues, including nutrient absorption, pest resistance, and shorter harvest times [2]. To mitigate these limitations, molecular tools such as genetic selection, mutagenic breeding, functional genomics, and advanced genome-editing technologies such as CRISPR/Cas have facilitated precise crop improvements [3] (Table 1). Consequently, there is an urgent need for more efficient breeding tools to overcome these barriers and accelerate the development of high-performing legume cultivars [4,5,6].

New technologies, such as haploid induction systems (HIS) and genome editing techniques, are being explored to tackle the challenges in legume breeding. A foundational step in both conventional and hybrid breeding is pure line selection, as pure lines are essential as parental stock for hybrid development [7]. During meiosis in higher plants, male and female gametes are produced, each containing half the number of chromosomes found in somatic cells. A haploid plant, which develops from a gametophyte without fertilization, possesses only a single set of chromosomes. Since haploid plants are typically sterile, chromosome doubling is necessary to generate stable and uniform offspring. This haploid induction process serves as a powerful tool in plant breeding, facilitating the rapid generation of genetically uniform doubled haploid (DH) lines [8,9]. Haploid breeding is advantageous as it accelerates the development of homozygous lines, typically achieved in just two generations, compared to the 6–8 generations required by traditional self-pollination methods. By doubling the haploid genome, breeders can create homozygous, genetically stable, and uniform plants, significantly reducing the time and labor required to develop pure lines. This technique is increasingly utilized in various fields, including horticulture, mutant isolation, and the whole-genome sequencing of naturally heterozygous species [10,11]. Haploid inducers help overcome genetic bottlenecks by enabling the faster development of homozygous lines, while genome editing tools such as CRISPR/Cas9 provide precise, targeted modifications to specific genes [12]. These innovations are revolutionizing our understanding of plant genetics and transforming the breeding process, allowing for more rapid and accurate enhancements in key traits such as yield, disease resistance, and stress tolerance. Consequently, these technologies are essential for the future of agricultural research, particularly in addressing the needs of global food systems. This review examines the potential of genome editing-mediated HISs in legumes, with a focus on recent advancements and ongoing challenges. Specifically, it highlights the role of cutting-edge technologies such as CRISPR/Cas9 and CRISPR/Cas12 in transforming legume breeding. The review aims to demonstrate how these tools can enhance key traits, including productivity, disease resistance, and environmental resilience. Furthermore, it discusses the integration of haploid induction and genome editing to accelerate the development of high-yielding, resilient legume varieties, thereby addressing global food security and sustainability challenges.

## 2. Haploid Induction in Legume Crops

**Figure 1:**
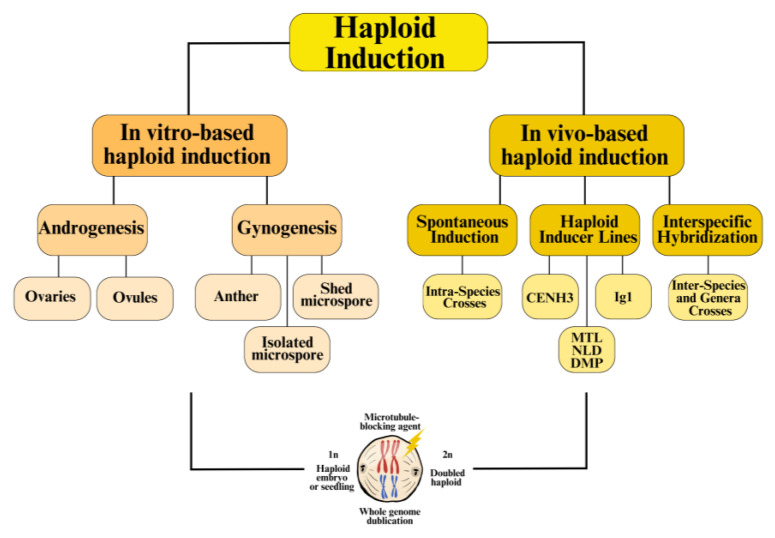
Overview of haploid induction methods in plants. Haploid induction can be divided into in vitro (gynogenesis and androgenesis) and in vivo methods (spontaneous induction, HILs, and interspecific hybridization). Microtubule-blocking agents disrupt spindle fiber formation during cell division, leading to whole-genome duplication. This process converts haploid cells into DH plants by preventing chromosome segregation, resulting in diploid cells.

Legumes, characterized by their genetic diversity and complex genomes, have historically posed challenges for haploid production [5,6]. However, recent advancements, including spontaneous haploid production, tissue culture techniques, and distant hybridization, have paved the way for more efficient breeding (Figure 1). Although these methods have their limitations, they establish a foundation for integrating modern tools such as haploid inducer lines (HIL) and genome editing technologies to accelerate crop improvement and enhance key traits in legume species.

### 2.1. Spontaneous Haploid Production

In certain plant species, haploids can spontaneously arise due to abnormalities in the development of reproductive organs, often resulting from male or female sterility. These natural disruptions in pollen or ovule development lead to the formation of haploid plants. Such spontaneous haploid production has been documented in over 400 species, including economically significant crops such as maize (*Zea mays*) and tobacco (*Nicotiana tabacum*). In maize, for instance, the *ig1* gene has been identified as a key factor in inducing haploid embryo formation from sperm cells [13]. Although spontaneous haploid production occurs naturally, its frequency is typically low and inconsistent, rendering it impractical for large-scale agricultural applications. This is particularly true for many legume species, where naturally induced haploids are rare, thus limiting the potential of this breeding method. In legumes such as alfalfa (*Medicago sativa*), often used as a model for genetic research, spontaneous haploid production has been demonstrated under controlled conditions. For example, Saunders and Bingham (1972) showed that alfalfa regenerants could be successfully obtained by culturing immature ovules in vitro [14]. However, even in these instances, the process remains inefficient and requires optimization to become feasible for practical breeding applications. While spontaneous haploid production presents an intriguing biological phenomenon, its low success rates and limited applicability in many legume species make it less reliable compared to alternative methods of haploid production, such as those involving haploid inducers or chemical treatments.

### 2.2. Tissue Culture Techniques

Tissue culture techniques for generating haploid plants, including gynogenesis and androgenesis, serve as powerful tools in crop breeding, facilitating the development of genetically uniform lines and accelerating the production of homozygous plants [9]. Gynogenesis is a process in which fertilization occurs, but the paternal genome is excluded from embryo development, resulting in a haploid plant derived solely from maternal genetic material [15]. This method can be induced in vitro, offering advantages such as shorter breeding cycles and the faster generation of homozygous lines, which are crucial for hybridization and genetic studies. Successful gynogenesis requires the optimization of factors such as the parental genotype, culture medium composition, and the developmental stage of the female gametophyte (ovule or embryo sac) [16]. While this approach has proven successful in species such as chickpeas (*C. arietinum*), its application in legumes is often limited by constraints related to reproductive biology and the challenges of inducing gynogenesis under controlled in vitro conditions. In soybean species, the complexity of ovule development, and the larger, more intricate genome, further complicate the process. Maternal effects and genotype–environment interactions also present challenges; however, ongoing research aims to refine these techniques for broader application in legume breeding [5,16,17].

Conversely, androgenesis involves the development of a haploid embryo from male gametes (microspores or anthers) without fertilization, resulting in a plant controlled solely by paternal genetic material [18]. This technique has been successfully applied to various crops, including maize, tobacco, and wheat (*Triticum aestivum*), and was first demonstrated in legumes such as alfalfa, where androgenesis was induced via anther culture in the 1980s [19]. Other leguminous species, including pea (*P. sativum*) [20] and clover (*Trifolium* spp.) [21], have also shown some success with androgenic methods, although overall success rates remain low. In legumes such as soybeans, androgenic induction is hindered by high paternal genome elimination during microspore or anther culture, along with challenges related to microspore development, genotype variability, and culture medium optimization. Despite these difficulties, advances in controlling environmental factors such as temperature, light, hormonal regimes, and genetic improvements hold promise for enhancing androgenic responsiveness in legumes, potentially accelerating the development of uniform, high-yielding cultivars [22,23].

### 2.3. Distant Hybridization-Induced Haploids

Distant hybridization-induced haploid production relies on the phenomenon of chromosome elimination, which occurs during interspecies hybridization. In this process, double fertilization leads to the formation of a zygote, but as the zygotic cells divide, paternal chromosomes may be selectively eliminated, resulting in haploid embryos. Additionally, rapid endosperm development can also contribute to the elimination of paternal chromosomes, often leading to seed abortion [24]. However, in some cases, the in vitro culture of hybrid embryos can rescue these haploids, allowing them to develop into viable plants. This technique has been successfully demonstrated in several crop species. For example, in the 1970s, researchers discovered that hybrids between cultivated barley (*Hordeum vulgare*) and *H. bulbosum* could produce haploid embryos through chromosome elimination [24]. Similarly, wheat and maize have been hybridized, with maize chromosomes being eliminated, followed by embryo culture and chromosome doubling to generate DH lines in wheat [5,25]. These systems have been effectively applied in barley, wheat, and other cereals to accelerate breeding.

However, the application of distant hybridization-induced haploids in legumes has proven more challenging [26]. While distant hybridization can theoretically induce haploid production in legumes, several factors limit the success of this approach. First, legume species often exhibit complex reproductive behaviors and strong genetic barriers between species, making hybridization difficult or unsuccessful. Additionally, the efficiency of chromosome elimination in legumes tends to be much lower than in cereal crops, further hindering the practical use of this technique [27].

In soybeans and other legumes, attempts at distant hybridization have yielded mixed results. Some hybridizations between common bean (*Phaseolus vulgaris*) and sweet pea (*Lathyrus odoratus*) have shown potential for inducing haploids through chromosome elimination, but the process remains inefficient [28]. Similarly, in pea (*P. sativum*), while distant hybridization with faba bean (*Vicia faba*) has been attempted, low success rates and difficulties with embryo rescue have rendered this approach largely impractical for accelerating breeding cycles in legumes [29].

One major challenge with legumes is that hybridization between species with distant genetic backgrounds often results in sterility or a poor seed set, complicating the production of viable haploid embryos. Unlike cereal crops, where hybridization-induced haploids are more frequently successful due to more compatible reproductive systems and better-established methods for embryo rescue, legumes often require specialized approaches to overcome these barriers [27]. Consequently, distant hybridization-induced haploids have not yet become a standard method for legume breeding. However, research into establishing legume-specific HILs, similar to those developed in cereals, may offer a more viable solution. By identifying specific genes or mechanisms that facilitate chromosome elimination in legumes, breeders could potentially create more efficient systems for haploid induction, thereby shortening breeding cycles and enabling more precise genetic improvements [4].

## 3. Haploid Inducer Lines in Legume Breeding

HILs are vital in modern plant breeding, enabling the rapid production of haploids that can be doubled to create pure, homozygous lines. This technology is particularly valuable for legumes, which are genetically complex and traditionally challenging to breed. HILs streamline the breeding process by bypassing self-pollination, significantly reducing the time required to develop new varieties [30,31]. When combined with genome editing tools such as CRISPR/Cas9, HILs facilitate precise trait improvements, accelerating enhancements in yield, disease resistance, and environmental resilience in legumes [12].

### 3.1. Mechanisms of Haploid Inducer Lines

HILs are widely utilized in modern breeding, enabling the rapid production of haploid plants that can be doubled to form homozygous diploids. HILs have been successfully applied in crops such as maize, wheat, and rice (*Oryza sativa*), stabilizing haploid lines in as few as two generations, thereby improving breeding efficiency and genetic quality [7]. Maize primarily contains two internal HILs: haploid induction based on the *indeterminate gametophyte* 1 (*ig*1) mutant and haploid induction based on the maize inbred line Stock6. The *ig1* mutant, found in the inbred line Wisconsin-23 (W23), can produce a haploid induction rate (HIR) of 3% when used as a parent. The resulting haploid from this inducible line contains the mother's cytoplasm and the male gamete's genome [32]. Although *ig1* mutants exhibit haploid induction effects, they also impact the formation of female gametes, disrupting nuclear division cycles and tubulin expression in female gametophytes. This leads to abnormal mitosis in some cells of the *ig1* embryo sac, resulting in an abnormal embryo sac structure, which often causes early seed abortion [32,33,34]. Importantly, the specific molecular mechanism by which *ig1* mutants induce haploid production remains unclear, limiting the application of *ig1* HIL.

Various genes mediate haploid induction through distinct molecular mechanisms. Extensive research has focused on haploid induction mechanisms in maize, particularly due to the discovery and application of the natural HIL Stock6. Gene mapping has identified at least seven possible quantitative trait loci (QTLs) involved in the haploid induction function of maize Stock6 [35,36,37]. Key genes such as *MATRILINEAL* (*MTL*), *PHOSPHOLIPASE A1* (*PLA1*), *NOT LIKE DAD* (*NLD*), and *DOMAIN OF UNKNOWN FUNCTION 679 membrane protein* (*DMP*) have been linked to haploid induction through phospholipase pathways. The *DMP* gene has broader applications in dicotyledonous plants but often exhibits a lower HIR. However, combining mutations in two genes has been shown to increase the HIR compared to single-gene mutations. Recent studies on another haploid induction gene, *PHOSPHOLIPASE D3* (*PLD3*), indicate that its mutation can increase the HIR threefold in the presence of the *mtl*/*zmpla1*/*nld* mutant line [37,38,39]. Kelliher et al. (2017) demonstrated that the MTL/PLA protein in maize targets the inner membrane of sperm cells, while the truncated protein in HILs fails to localize correctly, potentially affecting sperm cell membrane integrity and signaling pathways [35]. The transcriptome analysis of pollen RNA during haploid induction has revealed the overexpression of the pollen-specific genes involved in membrane composition, lipid metabolism, and calcium signaling, suggesting a connection to haploid induction [4,40].

CENH3 (centromere-specific histone H3) protein, featuring a highly conserved C-terminal histone fold domain (HFD) and a highly variable N-terminal tail, plays a critical role in loading CENH3 during meiosis and regulating cell division [41]. The sequence of its N-terminal domain varies significantly across different plants. Errors in the transcription, translation, modification, or incorporation of CENH3 may disrupt the assembly of the complete kinetosome, potentially leading to abnormal centromere function or inactivation [42,43,44,45,46]. Notably, the hybridization of N-terminally altered CENH3 with the wild type as a maternal parent produces haploids in *Arabidopsis* with an HIR of 25–45%. This strategy also was proven to be effective in wheat and maize, yielding HIRs of 8% and 0.86%, respectively [44,47,48,49]. Point mutations in the conserved C-terminal HFD of AtCENH3 result in haploid rates of 0.61–44% [12,50]. Studies indicate that damaged AtCENH3 with altered N-terminal or C-terminal point mutations cannot be loaded onto the centromere from the HIL parent in the zygote, leading to weakened centromere function and single-parent elimination [51]. Remarkably, heterozygous *+*/*cenh3* in maize resulted in 5% haploid production when outcrossed as a mother, while this proportion was only 0.4% in *Arabidopsis* [51,52]. The specific degradation of maternal EYFP-labeled CENH3 by nanoantibody targeting also resulted in haploid production [53]. These findings suggest that the amount of CENH3 on the maternal centromere is critical for maintaining the integrity of the maternal genome in offspring and can be leveraged for haploid breeding [54].

### 3.2. Development of Haploid Inducer Lines in Legumes

The application of HILs in legumes is still in its early stages, primarily due to the complex reproductive systems and genetic barriers characteristic of these crops. Unlike monocots, many legumes exhibit strong self-incompatibility and hybridization barriers, complicating the development of effective HILs [55]. Additionally, the HIR in dicots, including legumes, tends to be lower, further hindering their use in breeding programs. Despite these challenges, recent research has shown promise in developing legume-specific HILs. In soybeans, modified protocols derived from cereal crops have led to some success, although the HIR remains low [56]. Efforts with other legumes, such as *M. truncatula* [57], have encountered similar barriers, including difficulties with hybridization and low success rates.

Integrating genome editing tools such as CRISPR/Cas9 presents a potential breakthrough, allowing for targeted mutations in genes involved in gametophyte development [35,57,58,59]. When successfully integrated into breeding programs, HILs could provide significant advantages, such as accelerating the development of homozygous lines for traits such as disease resistance, drought tolerance, and improved nutritional content. For instance, in soybeans, HILs could reduce the time required to generate DH lines from 6–8 generations of self-pollination to just two generations, thereby speeding up the development of high-yielding, stress-resistant cultivars. However, to fully realize the potential of HILs in legume breeding, challenges such as low HIR and hybridization barriers must be addressed. Advances in genetic engineering, genome editing, and tissue culture protocols will be crucial for overcoming these hurdles and unlocking the benefits of HILs for legume crop improvement.

## 4. Genome Editing-Mediated Haploid Inducer System

### 4.1. Advances of Haploid Inducer Lines in Plants

The combination of HILs with genome editing technologies, such as CRISPR/Cas9 and CRISPR/Cas12, has the potential to streamline the breeding process, particularly for complex crops such as legumes [7,60]. This approach enables efficient haploid induction alongside precise gene editing, accelerating the development of homozygous lines and enhancing key traits such as yield, disease resistance, and stress tolerance. Traditional methods for generating homozygous lines are time-consuming and labor-intensive, typically requiring multiple generations of hybridization and selection. In contrast, genome editing, especially using CRISPR/Cas technologies, offers a more efficient and precise alternative. CRISPR/Cas constructs can be introduced early during fertilization to target and edit the recipient plant genome while eliminating the haploid inducer's genome. This results in a haploid plant that, following chromosome doubling, becomes a non-transgenic homozygous edited plant [61,62]. This strategy significantly accelerates the development of plants with desired traits by reducing the number of generations needed to achieve homozygosity.

CRISPR/Cas9 technology has been effectively utilized in maize to enhance haploid induction efficiency. Jiang et al. (2021) demonstrated that knocking out the *ZmPLD3* gene boosted the HIR from 1% to 4% in the *mtl*/*zmpla1*/*nld* mutant line, showcasing the potential of genome editing to improve haploid induction, a persistent challenge in legumes [57]. Additionally, the *ZmPOD65* (*Peroxidase 65*) gene in maize, which enhances haploid induction via chemical pollen treatment, could be combined with genome editing to optimize haploid induction in legumes, where natural haploid induction is still limited [37,63]. The CRISPR/Cas12 (Cpf1) system provides another valuable tool for gene editing. It recognizes a different protospacer adjacent motif (PAM) sequence and generates sticky ends, offering more efficient editing with fewer off-target effects compared to CRISPR/Cas9. By introducing CRISPR/Cas12 constructs into HILs and crossing them with elite inbred lines, edited haploids can be obtained and doubled to form homozygous diploid plants.

Incorporating gene-editing technology into HILs simplifies the breeding process, reducing the time required to achieve homozygosity compared to traditional methods that involve laborious hybridization and selection [64,65]. This method accelerates the breeding cycle and can enhance key traits such as yield, disease resistance, and stress tolerance in legume crops such as soybeans, chickpeas, and peas [64]. Table 2 summarizes the application of genome editing in haploid development across various crops.

Integrating genome editing with haploid induction presents a promising strategy for addressing the challenges inherent in legume breeding. However, significant obstacles remain in effectively applying these technologies to legumes. One major challenge is the relatively low HIR observed in many legume species, compounded by the genetic complexity of their genomes. Establishing effective HILs in legumes is still in the early stages of research [77,78,79,80,81]. While progress has been made, optimizing genome editing and HILs tailored to legumes will be crucial for unlocking their full potential. Developing genome-edited HILs specific to legumes is essential for overcoming current limitations and accelerating the creation of homozygous, high-performance legume cultivars [82,83,84]. As these technologies continue to evolve, they hold the potential to transform legume breeding, thereby improving food security and agricultural sustainability.

### 4.2. Genome Editing-Mediated Haploid Inducer Systems in Legumes

Integrating HILs with genome editing technologies holds immense potential for transforming legume breeding. The combination of haploid induction and gene editing creates new opportunities for improving breeding efficiency, enhancing desirable traits, and lowering production costs in legume crops. Recent advances in molecular breeding have shown that gene editing can effectively enhance specific genes and produce haploid progeny through hybridization with wild types, offering significant cost savings and broad application potential in molecular breeding [85,86]. For instance, mutations in *DMP* homologs have induced haploid production during seed development in several legumes. In maize, mutations in *MTL*/*NLD*/*PLA1* have significantly improved haploid induction [35,39,40]. 

**Figure 2:**
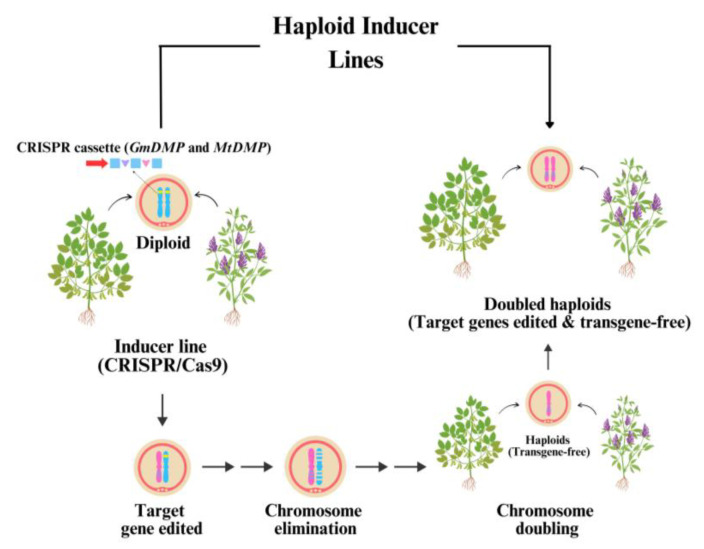
Potential application of candidate *DMP* genes for haploid induction in legumes. CRISPR/Cas9-mediated candidate *DMP* genes (e.g., *GmDMP* and *MtDMP*) are used in HILs to enable chromosome elimination, producing haploids that undergo chromosome doubling to form DHs. These transgene-free, gene-edited plants serve as valuable tools for legume breeding programs.

While *MTL*/*NLD*/*PLA1* is not conserved in dicots, *DMP* genes are conserved across monocots and dicots (including legumes). The functional loss of *DMP* homologs triggers maternal haploid induction in *Arabidopsis* [4,35], suggesting that *DMP*-triggered in vivo HISs could be applied to legumes (Figure 2). Homologs of *DMP* have been identified in several legumes, including soybean and alfalfa [56,57], laying the groundwork for legume haploid breeding systems. Successful instances of haploid induction in soybean and alfalfa have showcased the potential for the rapid development of stable homozygous lines. In soybeans, haploid induction has facilitated bypassing several generations of traditional breeding, accelerating the creation of high-performing cultivars. This improved breeding efficiency is vital for meeting the increasing demand for legumes, especially in light of climate change and environmental stressors that affect crop productivity.

### 4.3. Challenges and Limitations

While HISs hold great potential to accelerate legume breeding, several challenges must be addressed before widespread adoption. Key obstacles include a low HIR, technical issues with genome editing, concerns over genetic diversity, and regulatory hurdles. The large and complex genomes of legumes complicate haploid induction and regeneration, necessitating specialized agronomic measures and expertise. Additionally, legume species such as soybean and chickpea exhibit low transformation efficiencies, which limits the success of haploid induction methods. Although anther and pollen cultures can shorten breeding cycles and enhance selection efficiency, they often suffer from low embryo viability and early embryo abortion. Therefore, developing stable and efficient haploid induction techniques remains a critical bottleneck in legume breeding [57]. Anther or pollen culture is particularly inefficient in crops such as soybeans due to microspores failing to undergo the necessary developmental stages for haploid embryo formation. Early-stage embryo abortion further reduces success rates, and while embryo culture can sometimes rescue embryos, the process remains labor-intensive and prone to failure [56,57].

While haploid induction accelerates the generation of homozygous lines, it also poses a risk to genetic diversity. Traditional breeding methods maintain genetic variation, which is essential for adapting crops to environmental changes and combating diseases. However, generating pure lines in just two generations may narrow the genetic base, especially if only a few elite lines are crossed [22]. This can lead to inbreeding depression or reduced adaptability. To ensure long-term genetic improvement, it is crucial to integrate diverse genetic sources when employing HISs.

### 4.4. Future Prospects

The future of HISs in legume breeding holds significant promise, particularly in addressing challenges related to climate change, population growth, and food insecurity. These issues underscore the urgent need for faster, more efficient breeding methods to enhance crop yield, disease resistance, and environmental resilience. Haploid breeding can rapidly stabilize beneficial traits and eliminate deleterious alleles, positioning it as a vital tool for legume crop improvement in the coming decades. However, overcoming the current limitations is essential to fully realizing its potential.

While HILs have been successful in monocots such as maize and wheat, their application in legumes remains underdeveloped. A major future focus will be the creation of more efficient HILs tailored to legume species. This will require identifying genetic loci and molecular pathways that facilitate haploid induction in legumes. The growing demand for DH lines necessitates low-cost, large-scale production systems. Integrating data analysis technologies could enable high-throughput DH evaluation and create a model for intelligent breeding. 

Identifying legume-specific HILs could help address current limitations, such as low HIR. Incorporating wild relatives or exotic germplasm with high haploid induction potential may expand the genetic pool. Furthermore, optimizing tissue culture protocols to improve the survival and regeneration of haploid embryos in species such as soybeans, chickpeas, and lentils will be key to increasing success in embryo rescue and regeneration [87].

## 5. Conclusions

Legumes are vital to sustainable agriculture, providing essential plant-based proteins, enhancing soil health through nitrogen fixation, and increasing farm productivity. Genome editing enabled by haploid inducers has the potential to revolutionize legume breeding by facilitating the rapid development of elite varieties with improved traits. Ultimately, the combination of genome editing and HISs offers significant promise for creating more sustainable and resilient agricultural systems, thereby addressing the nutritional needs of a growing global population.

## Figures and Tables

**Table 1:** Comparison of conventional and modern plant breeding methods.

Conventional Plant Breeding	Modern Plant Breeding
Phenotype selection, leading to less accuracy	Genotype and phenotype selection, higher accuracy
Slow to develop and release new varieties	Faster development of new varieties
Relies on hybridization for various varieties	Uses advanced tools such as genomic selection and high-throughput phenotyping (HTP)
Recessive alleles take longer	Efficiently incorporates recessive alleles using markers
Depends on the breeder's skill, often inconsistent	Based on scientific data, making it more producible
Requires minimal technical expertise	Requires advanced technical and genetic knowledge
Low cost due to basic tools	High cost due to advanced technologies

**Table 2:** Summary of the genome editing-mediated HILs using CRISPR/Cas9.

Plant Species	Gene Editing Method	References
*Arabidopsis thaliana*	*CENH3*, *GFP-CENH3-tailswap* (N-terminal fusion green fluorescent protein-tagged) *CENH3*, *CENH3*-tailswap (tail switching)	[47]
	*CENH3* point mutation	[50,66]
	*BrCENH3*/*LoCENH3* complement *AtCENH3* (heterologous complementation and N-terminal heterologous exchange)	[44]
	*AtDMP9* knockout, *Atdmp*/*Atdmp*	[4]
*Brassica napus*	*BnaDMP* knockout, *Bnadmp*/*Bnadmp*	[67,68,69]
*B. oleracea*	*BoC03.DMP9* knockout, *BoC03.dmp9*/*BoC03.dmp9*	[68]
*Citrullus lanatus*	*DMP* point mutation	[70]
*G. max*	*DMP* point mutation	[4]
*M. sativa*	*MtDMP* knockout, *Mtdmp*/*Mtdmp*	[57]
*N. tabacum*	*NtDMP* knockout, *Ntdmp*/*Ntdmp*	[71]
*O. sativa*	*OsPLA1* knockout, *Ospla1*/*Ospla1*	[72]
*T. aestivum*	*CENH3* knockout, *+*/*Tacenh3α*	[49]
	*CENH3* knockout, *Tamtl*/*Tamtl*	[60]
*Solanum lycopersicum*	*SlDMP* knockout, *Sldmp*/*Sldmp*	[73]
*S. tuberosum*	*StDMP* knockout, *Stdmp*/*Stdmp*	[73]
*Z. mays*	*CENH3*, N-terminal modification	[48]
	*ZmPLA1* knockout, *Zmpla1*/*Zmpla1*	[74]
	*CENH3* knockout, *+*/*cenh3*	[52]
	*ZmPLD3* knockout, *Zmpld3*/*Zmpld3*	[70]
	*ZmKNL2* knockout, *Zmknl2*/*Zmknl2*	[75]
	*ZmPOD65* knockout, *Zmpod65*/*Zmpod65*	[63]
	*ClDMP4* knockout, *Cldmp4*/*Cldmp4* *CENH3* point mutation	[63]
	*MTL*/*NLD*/*ZMPLA1* point mutation	[40,76]

Note: The table includes key genes involved in haploid induction, listed alphabetically as follows: CENTROMERIC HISTONE H3 (CENH3); DOMAIN OF UNKNOWN FUNCTION 679 membrane protein (DMP); KINETOCHORE NULL 2 (KNL2); MATRILINEAL (MTL); NOT LIKE DAD (NLD); PEROXIDASE (POD65); PHOSPHOLIPASE A1 (PLA1); and PHOSPHOLIPASE D3 (PLD3).

## Data Availability

Not Applicable.
